# Scleromyositis: A Novel Entity Beyond Systemic Sclerosis and Autoimmune Myositis

**DOI:** 10.7759/cureus.44292

**Published:** 2023-08-28

**Authors:** Osvaldo Alexis Marche Fernandez, Lizbeth Teresa Becerril Mendoza, Luis Jose Pinto Garcia, Jesus Guillermo Hernandez Garcia, Juan Daniel Diaz Garcia

**Affiliations:** 1 Internal Medicine, Hospital Juárez de México, Mexico, MEX

**Keywords:** limited systemic sclerosis, clinical rheumatology, inflammatory myositis, diffuse systemic sclerosis, scleromyositis

## Abstract

Scleromyositis is a new clinical entity, which not only has clinical and histopathological components of systemic sclerosis and inflammatory myopathy but is also characterized by presenting unique characteristics, which may not be in the previously mentioned diseases. Up until now, there are no specific classification criteria proposed by the American College of Rheumatology or the European League Against Rheumatism (ACR/EULAR). This paper presents a case report of a female patient in her 60s who was admitted to our institution due to muscle weakness in her legs and dysphagia. Within her diagnosis approach, clinical characteristics compatible with autoimmune myopathy were found; however, she presented with anti-PM/Scl75 antibody-positive results. In this paper, we emphasized the clinical characteristics and forms of presentation of scleromyositis, additionally discussing the available treatment for this entity.

## Introduction

Systemic sclerosis is a rheumatic disease that affects multiple systems and organs [1]. This clinical entity generally has a higher prevalence in females than in males, with a reported ratio of between 3:1 and 8:11. In some cases, this disease can present as an overlap syndrome, as reported in the meta-analysis of Muangchan et al. [2], with 13% of patients diagnosed with systemic sclerosis presenting with myositis or with a myopathy.

On the other hand, autoimmune myositis is a group of pathologies that are characterized by presenting muscle inflammation that leads to weakness and, in some cases, elevated muscle enzymes [3]. Like the previous entity, the prevalence of this pathology is higher in females than in males, with an estimated prevalence ratio of between 2.9 and 3.4 per 100,000 individuals [3]. The literature mentions that one of the presentations of autoimmune myositis is an overlap syndrome with other connective tissue diseases, with 40% of cases overlapping with systemic sclerosis [4]. The association of both clinical entities has generated a new pathology that must meet the classification criteria proposed by the American College of Rheumatology and the European League Against Rheumatism (ACR/EULAR) for both pathologies [4]. However, this condition is not the best diagnosis method. Therefore, the identification of new accurate clinical and histopathological criteria becomes a priority to avoid an unsuccessful diagnosis and treatment.

Scleromyositis is the name that has been proposed for this clinical entity that falls between the clinical spectrum of systemic sclerosis and autoimmune myositis [4,5]. Clinical manifestations are characterized by muscle weakness in up to 88% of cases, pulmonary in 68%, cardiovascular in 21%, dermatological in 40%, and gastrointestinal disease in approximately 65% [4].

Muscle biopsy represents one of the main diagnostic options alongside quantification of antibody levels [5]. Right now, the treatment of this entity remains controversial, since there are no clinical practice guidelines to support the best therapeutic management. From this point of view, steroids represent the first therapeutic line with the drawback of causing kidney damage [6]; subsequently, management with immunomodulatory drugs (mycophenolate mofetil) [7] is indicated. However, it is evident that a greater number of studies are needed to determine the best therapeutic management.

## Case presentation

A female in her 60s, with a medical history of antisynthetase and chronic kidney disease, went to the emergency department of our institution due to the presence of muscle weakness, with greater involvement of the pelvic extremities accompanied by unintentional weight loss and progressive dysphagia, initially consuming solid foods and later liquids. As a result of the physical examination, the patient presented with a blood pressure of 74/58 mmHg and a heart rate of 97 bpm. Then, the venous excess ultrasound score (VExUS) protocol was performed, finding data on fluid congestion, for which it was decided to start a vasopressor (norepinephrine). In the same way, the presence of muscular weakness was still there, both in the pelvic and thoracic extremities, quantified in 2/5, together with skin affection (lichenification) in both hands and forearms. Given the suspicion of systemic sclerosis due to skin, muscle, and gastrointestinal involvement, an antibody panel was requested, with a positive report of anti-PM/Scl75 and anti-MI-2β antibodies (Table 1), making the diagnosis of scleromyositis.

**Table 1:** Antibody panel *This table shows the antibodies that were requested from the patient, finding the anti-PM/Scl75 positive, ANA positive, and anti-MI-2β positive. ANA: antinuclear antibody, anti-dsDNA: anti-double-stranded DNA, anti-SM: anti-Smith, anti-SSa: anti-Sjögren’s syndrome-related antigen A, anti-SSb: anti-Sjögren’s syndrome-related antigen B

Antibody	Result
*Anti-PM/Scl75	12 (positive)
*Anti-MI-2β	12 (positive)
*ANA	1:160 homogeneous nuclear pattern
Anti-B2	<2 (negative)
Anti-dsDNA	<10 (negative)
Anti-SM	<2 (negative)
Anti-SSa	<2 (negative)
Anti-SSb	<2 (negative)
Anti-Jo-1	1 (negative)
Anti-PM/Scl100	2 (negative)
Anti-Ku	1 (negative)

Investigations

**Figure 1:**
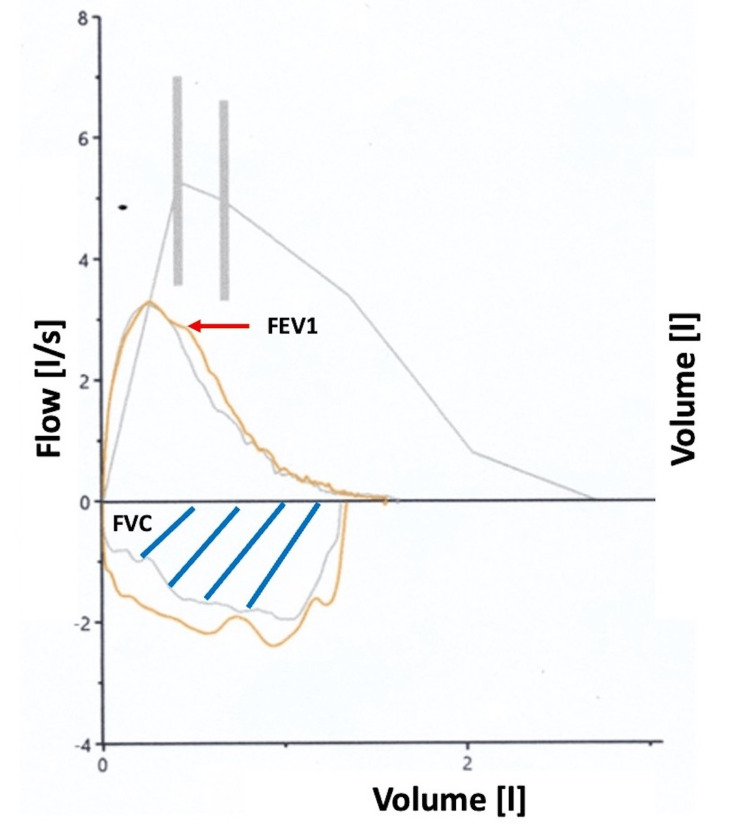
Flow-volume curve showing a decrease in FVC (blue lines showing the area under the curve) by 59% and FEV1 (red arrow) by 53%; a moderate restrictive pattern is then established FVC: forced vital capacity, FEV1: forced expiratory volume in one second

During the patient’s hospitalization, as part of the diagnostic approach, a simple and contrast-enhanced tomography of the thorax, abdomen, and pelvis was performed by the internal medicine department. The presence of bilateral pleural effusion together with passive atelectasis was reported. However, there was no evidence of interstitial or diffuse lung damage. Subsequently, spirometry was elaborated, in which it reported a restrictive pattern without changes, with the use of a bronchodilator (Figure 1). The diffusing capacity of the lung for carbon monoxide (DLCO) of the patient was 55%.

Serum creatine phosphokinase (CPK) levels were normal (72 UI/L). On the other hand, in the endoscopy study together with esophagus manometry, there was a presence of ineffective esophageal motility by Chicago 4 classification. Therefore, immunosuppressive management (prednisone together with azathioprine) was started, with which the patient presented clinical improvement but still with a decrease in muscle strength without elevation of muscle enzymes. Consequently, an electromyography was performed in conjunction with a sensory and nerve conduction study. The results showed the existence of a severe motor polyneuropathic involvement of an axonal nature with secondary demyelinating changes that involved the nerves of the four extremities. For this reason, physical rehabilitation therapy was started.

Treatment

After the diagnosis of scleromyositis, management was started with prednisone calculated at 1 mg/kg of weight, together with immunomodulatory therapy with azathioprine calculated at a dose of 1 mg/kg. As part of the comprehensive management of the patient, physical and muscular rehabilitation therapy was also included. Initially, there was improvement in the symptoms; however, later, the patient presented deterioration, with greater muscle weakness and progression of dysphagia. Given this situation, it was decided to change the prednisone to hydrocortisone at a dose of 40 mg every eight hours (equivalent to 30 mg of prednisone).

Outcome and follow-up

The patient died 24 days after the hospital stay. Specifically, 48 hours after performing the esophagus manometry, the patient exhibited greater neurological deterioration, with decreased muscle strength and loss of control of the bladder sphincter, presenting acute urinary retention that leads to the placement of a urethral catheter. Subsequently, she began with the presence of fever, in addition to elevated leukocytes and neutrophils, for which we took a general examination of urine, urine culture, blood cultures, and chest X-ray. Forty-eight hours after taking the urine culture, extended-spectrum beta-lactamase (ESBL) *E. coli* 100,000 colony-forming units were reported. Therefore, antimicrobial management with ertapenem was started. Hemodynamic and renal deterioration occurred 24 hours later, leading to an infusion of norepinephrine at 0.16 μg/kg/minute along with a continuous renal replacement therapy session. However, the patient died 48 hours later.

## Discussion

Scleromyositis is a disease that involves more than the overlap syndrome between autoimmune myositis and systemic sclerosis [4]. As illustrated in the clinical case, its form of clinical presentation is varied, with predominant symptoms and signs involving the musculoskeletal system (mainly muscle weakness) [4]. As a systemic disease, other organs are involved; this can be seen in the randomized clinical study proposed by De Lorenzo et al. [8], which demonstrated that patients with anti-PM/Scl antibodies could present with dermatological manifestations such as Raynaud’s phenomenon, telangiectasias, skin ulcers, lichenification, livedo reticularis, and edema subcutaneous. Also, gastrointestinal (dysphagia and gastroesophageal reflux) and systemic (fever, pericarditis, and glomerulonephritis) manifestations are described [8]. Likewise, different studies [4-8] emphasize that lung disease is one of the most important and significant manifestations, having an impact on the deterioration of the patient’s life quality. Interstitial lung disease represented the leading cause of death (33% of cases) in the European Scleroderma Trials and Research (EUSTAR) cohort [9], and it has also been described that it can occur in 40% of patients with systemic sclerosis [4]. The identification of lung damage must be carried out by means of high-resolution tomography; however, tests such as spirometry and the diffusing capacity of the lung for carbon monoxide (DLCO) can be useful [10].

Currently, there are no histological criteria to determine the muscular condition of scleromyositis. However, in the study proposed by Lefebvre et al. [5], the muscle biopsy performed on patients with systemic sclerosis plus an associated autoimmune myositis showed that in more than 50% of the cases, there were inflammatory infiltrates, which were mostly made up of T lymphocytes (49%), with the presence of CD4+ (32%) and CD8+ (22%). On the other hand, electromyography generally shows a neuropathic pattern with greater proximal than distal involvement, described in 18%-29% of patients with scleromyositis [4].

The detection of autoantibodies is another criterion for the diagnosis of scleromyositis. However, those presented in systemic sclerosis (anti-centromeres, anti-topoisomerase I, and anti-RNA polymerase III) are not specific for scleromyositis. Anti-PM/Scl antibodies are the most frequently presented (31%), followed by anti-Ku (38%-55%), and anti-U1RNP (10%-46%) [4].

Regarding treatment, there are no specific clinical practice guidelines to regulate the medical management of this disease, so it is currently recommended to start its management with steroids. In patients with positive anti-PM/Scl antibodies, there is greater efficacy than in those without them. It has been documented that the use of mycophenolate mofetil for 24 months or cyclophosphamide for 12 months improves lung function in systemic sclerosis, but there is a lack of evidence with the use of other therapies [11].

Learning points

Scleromyositis is a unique disease that is not the conjunction of systemic sclerosis and autoimmune myositis, but the presence of systemic symptoms and musculoskeletal manifestations.

Anti-PM/Scl antibodies represent the most common autoantibody in scleromyositis. As mentioned above, it is necessary to establish clinical, biochemical, and histopathological criteria for the diagnosis of this disease to identify in an easier way patients with clinical characteristics like our patient.

At the moment, treatment with steroids and immunomodulatory drugs (azathioprine, mycophenolate mofetil, and cyclophosphamide) are the best options for the management of this disease. However, more randomized clinical trials are needed to establish better therapeutic options for this disease.

## Conclusions

In conclusion, more clinical studies are necessary to establish the diagnostic criteria for this disease, which is beginning to appear more frequently in our setting, in order not only to better understand its symptoms but also to determine the best therapeutic options.
